# Air Exposure Induced Characteristics of Dry Eye in Conjunctival Tissue Culture

**DOI:** 10.1371/journal.pone.0087368

**Published:** 2014-01-31

**Authors:** Hui Lin, Yangluowa Qu, Zhixin Geng, Cheng Li, Huping Wu, Nuo Dong, Zuguo Liu, Wei Li

**Affiliations:** 1 Eye Institute of Xiamen University, Xiamen, Fujian, China; 2 Xiamen University affiliated Xiamen Eye Center, Xiamen, Fujian, China; 3 Fujian Provincial Key Laboratory of Ophthalmology and Visual Science, Xiamen, Fujian, China; Wayne State University, United States of America

## Abstract

There are several animal models illustrating dry eye pathophysiology. Current study would like to establish an ex vivo tissue culture model for characterizing dry eye. Human conjunctival explants were cultured under airlift or submerged conditions for up to 2 weeks, and only airlifted conjunctival cultures underwent increased epithelial stratification. Starting on day 4, the suprabasal cells displayed decreased K19 expression whereas K10 keratin became evident in airlift group. Pax6 nuclear expression attenuated already at 2 days, while its perinuclear and cytoplasmic expression gradually increased. MUC5AC and MUC19 expression dramatically decreased whereas the full thickness MUC4 and MUC16 expression pattern disappeared soon after initiating the airlift condition. Real time PCR showed K16, K10 and MUC16 gene up-regulated while K19, MUC5AC, MUC19 and MUC4 down-regulated on day 8 and day 14. On day 2 was the appearance of apoptotic epithelial and stromal cells appeared. The Wnt signaling pathway was transiently activated from day 2 to day 10. The inflammatory mediators IL-1β, TNF-α, and MMP-9 were detected in the conditioned media after 6 to 8 days. In conclusion, airlifted conjunctival tissue cultures demonstrated Wnt signaling pathway activation, coupled with squamous metaplasia, mucin pattern alteration, apoptosis and upregulation of proinflammatory cytokine expression. These changes mimic the pathohistological alterations described in dry eye. This correspondence suggests that insight into the pathophysiology of dry eye may be aided through the use of airlifted conjunctival tissue cultures.

## Introduction

Dry eye is one of the most prevalent ocular surface disorders, affecting about 5% to over 35% of the adult population at various ages [1]. It is a not fully understood complex condition having different causes that include lacrimal gland insufficiency, meibomian gland dysfunction, impairment of neuronal innervation, and environmental stress [2]. The common dry eye ocular surface epithelial histological hallmarks include abnormal proliferation and differentiation, apoptosis, epithelial barrier function breakdown, decreased density of conjunctival goblet cells, inflammatory cell infiltration, and decreased as well as altered production of mucin expression patterns [3], [4].

Several animal models have been developed to simulate the pathophysiologic changes occurring in dry eye [2], [5]. For instance, desiccating stress and/or drug-induced decreases in tear fluid production in mice elicited typical ocular surface epithelial changes resembling human dry eye [6]–[9]. In these desiccating models, there was pronounced conjunctival epithelial cell layer stratification, goblet cell loss, and apoptosis as well as rises in matrix metalloproteinases (MMPs) production. Such changes suggest that these models are useful tools to study dry eye ocular surface pathology. Other animal models using instead transgenic mice [10], rat [11], rabbit [12]–[14], and dog [15], [16] have also provided understanding of different dry eye etiologies inducing specific pathophysiological changes. Other than animal models, the molecular events underlying some of the pathophysiological changes of dry eye have been delineated using immortalized human or mouse corneal and conjunctival epithelial cell lines [4], [17], [18].

Although animal models have provided important leads on understanding dry eye related changes within a sophisticated in vivo system, however, complexity of in vivo environment makes it difficult to delineate certain signaling pathway(s) involved in dry eye under well-defined conditions. From this point of view, ex vivo tissue culture may have advantage in decoding the pathological mechanism in dry eye. Our previous study established a squamous metaplasia model by culturing human limbal tissue at air-liquid interface (airlift) [19]. Since squamous metaplasia is commonly found in many ocular surface diseases manifesting dry eye, we hypothesized that airlift culture model is appropriate to dissect pathophysiological changes occurring in dry eye. As the conjunctiva constitutes majority of the ocular surface, and the conjunctival epithelium is populated by goblet cells, which are the primary source of secreted mucins in the tear film, it may be feasible to use this tissue to develop a model for dry eye. In this study, we showed that the pathological alterations in human conjunctival explants cultured at an air-fluid interface indeed mimic some of the changes described in dry eye.

## Methods

### Materials and Reagents

Dulbecco’s modified Eagle’s medium (DMEM), Ham’s/F12 medium, HEPES buffer, antibiotic/antimycotic mix, fetal bovine serum (FBS), epidermal growth factor (EGF), TRIzol, and Alexa Fluor® 488 goat anti-chicken IgG were from Life Technologies (Carlsbad, CA). Hydrocortisone, dimethyl sulfoxide, cholera toxin, insulin-transferrin-sodium selenite media supplement, 3% hydrogen peroxide, acetone, Triton X-100, bovine serum albumin (BSA), FITC conjugated anti-mouse, goat, and rabbit IgGs were from Sigma (St. Louis, MO). Mouse anti-cytokeratin 10 (K10), cytokeratin 19 (K19), and p63 antibodies, and diaminobenzidine (DAB) were from Dako Cytomation (Carpinteria, CA). Mouse anti-Pax6, cytokeratin 16 (K16) and β-catenin antibodies were from Santa Cruz Biotechnology (Santa Cruz, CA). Mouse anti-MUC5AC, MUC4, and MUC16 antibodies and FITC conjugated anti-chicken IgG were from Abcam (Cambridge, MA). Chicken anti-MUC19 antibody was kindly provided by Professor Chen Yin [20]. Rabbit anti-phosphor-β-catenin antibody was from Cell Signaling Technology (Danvers, MA). Immunofluorescence mounting medium with DAPI, mounting medium with propidium iodide, ABC kit (Vectastain Elite) for mouse IgG and ABC kit for rabbit IgG were from Vector Laboratories (Burlingame, CA). Type I collagen and 6 well plate insert were from BD Bioscience Corporation (San Jose, CA). DeadEnd fluorometric TUNEL system was from Promega (Madison, WI). Human MMP-9, IL-1β and TNF-α ELISA kits were from Boster (Wuhan, Hubei, China). DNeasy Blood & Tissue Kit was obtained from Qiagen (Valencia, CA). The ExScript RT Reagent kit and SYBR Premix Ex Taq Kit were obtained from Takara Bio (Shiga, Japan).

### Human Conjunctival Explant Cultures

The study was approved by the ethical committee of Xiamen University affiliated Xiamen Eye Center. Human tissue was handled according to the Declaration of Helsinki. Bulbal conjunctival tissues from human donor eyes without ocular surface disease history were obtained from the Eye Bank of Xiamen Eye Center (Xiamen, China). The tissue was rinsed three times with PBS containing antibiotic/antimycotic mix, then transferred to supplemented hormonal epithelial medium (SHEM) made of an equal volume of HEPES-buffered DMEM containing bicarbonate and Ham’s/F12, supplemented with 5% FBS, 0.5% dimethyl sulfoxide, 2 ng/ml mouse EGF, 5 µg/ml insulin, 5 µg/ml transferrin, 5 ng/ml selenium, 0.5 µg/ml hydrocortisone, 1 nM cholera toxin, and antibiotic/antimycotic mix. The conjunctival tissue was then cut into segments (explants) of 5×5 mm size or trephined into 7 mm in diameter. After that, conjunctival explants were placed on the center of 1 mg/ml type I collagen-coated inserts in 6-well plate containing SHEM.

Conjunctival explants were cultured in airlift or submerged conditions as previously reported [19]. Cultures were incubated at 37°C under 5% CO_2_ and 95% air, and the medium was changed every 2 days. For those trephined explants, conditioned media were collected, aliquoted and stored in −80°C freezer for ELISA assay. Explants in parallel experiments were terminated every 2 days for 2 weeks. Freshly isolated conjunctival tissue without culture was served as normal control.

### Histology and Immunostaining

Cryostat sections (6 µm) of the conjunctiva and conjunctival explants were fixed in acetone for 10 min at −20°C, and prepared for hematoxylin-eosin staining and immunostaining. For immunofluorescence staining, sections were rehydrated in PBS followed by incubation in 0.2% Triton X-100 for 10 min. After three rinses with PBS for 5 min each and preincubation with 2% BSA to block nonspecific staining, sections were incubated with primary antibodies for 1 h with different dilutions (K19, K10 and MUC5AC all at 1∶200, MUC16 and K16 at 1∶100, β-catenin and phosphor-β-catenin at 1∶150). After three washes with PBS for 15 min, they were incubated with an FITC-conjugated secondary antibody (goat anti-rabbit or anti-mouse IgG at 1∶100) for 1 h. After three additional PBS washes for 15 min, they were counterstained and mounted with mounting medium with DAPI/PI, and observed under a Nikon TE-2000 U Eclipse epi-fluorescence microscope (Nikon, Japan) or Olympus FluoView™ FV1000 confocal microscope (Olympus, Japan). For immunohistochemical staining, the endogenous peroxidase activity was blocked by 0.6% hydrogen peroxide for 10 min after fixation, while nonspecific staining was blocked by 1% normal serum for 30 min. Sections were then incubated with antibodies for 1 h with different dilutions (p63 at 1∶50, Pax6 at 1∶200, MUC4 at 1∶100). After three washes with PBS for 15 min, sections were incubated with biotinylated anti-mouse or anti-rabbit IgG for 1 h, followed by incubation with ABC reagent for 45 min, the reaction product was then developed with DAB for 2 min. Sections were photographed using a Nikon Digital Sight DS-Fi1 camera mounted on a Nikon Eclipse 50 i light microscope (Nikon, Japan).

### RNA Isolation and Real-Time PCR

RNA was isolated from explants on D0, D8 and D14 using TRIzol (Invitrogen) and reverse transcribed to cDNA using the ExScript RT Reagent kit. Real-time PCR was performed with a StepOne Real-Time PCR detection system (Applied Biosystems, Darmstadt, Germany) using an SYBR Premix Ex Taq Kit, according to the manufacturer’s instructions. The amplification program included an initial denaturation step at 95°C for 10 minutes, followed by denaturation at 95°C for 10 seconds, and annealing and extension at 60°C for 30 seconds, for 40 cycles. SYBR Green fluorescence was measured after each extension step, and the specificity of amplification was evaluated by melting curve analysis. The primers used to amplify specific gene products are shown in Table 1. The results of the relative quantitative real-time PCR were analyzed by the comparative CT method and normalized to β-actin as an internal control.

**Table 1:** Primer Sequence Pairs Used for Quantitative Real-Time PCR.

Gene	Sense	Antisense
β-actin	TGACGTGGACATCCGCAAAG	CTGGAAGGTGGACAGCGAGG
K16	ACCGAGGAGCTGAACAAAGA	GTTCTCCAGGGATGCTTTCA
K19	TTTGAGACGGAACAGGCTCT	AGCTCTTCCTTCAGGCCTTC
K10	AGCATGGCAACTCACATCAG	TGTCGATCTGAAGCAGGATG
MUC5AC	TCCACCATATACCGCCACAGA	TGGACCGACAGTCACTGTCAAC
MUC19	GGGTGCTTTTGTCCAGAAGG	TTGCAGCCAACAGAAGTGAC
MUC4	GCCCAAGCTACAGTGTGACTCA	ATGGTGCCGTTGTAATTTGTTGT
MUC16	GCCTCTACCTTAACGGTTACAATGAA	GGTACCCCATGGCTGTTGTG

### TUNEL Assay

Terminal deoxyribonucleotidyl transferase-mediated FITC-linked dUTP nick-end DNA labelling (TUNEL) assay was performed according to the manufacturer’s instructions. Briefly, sections were fixed in 4% formaldehyde for 20 min at room temperature and permeabilized with 1% Triton X-100, then incubated for 60 min at 37°C with exogenous TdT and fluorescein-conjugated dUTP for repair of nicked 30-hydroxyl DNA ends. Sample was treated with DNase I as the positive control, while the negative control was incubated with buffer lacking rTdT enzyme. The slides were mounted with DAPI mounting media to stain the nuclei.

### ELISA Assay

IL-1β, TNF-α, and MMP-9 content in the conditioned media was determined following centrifugation at 10,000×g for 10 min. It was done by using the supernatant which was subjected to enzyme-linked immuno-sorbent assay (ELISA) according to the manufacturer’s instructions. The optical absorbance was measured at 450 nm with a BioTek Elx800 microplate reader. Protein amounts in aliquots of conditioned media were calculated according to the standard curve generated from recombinant IL-1β, TNF-α and MMP-9 provided by the vendor. Total DNA was extracted by using DNeasy Kit according to the manufacturer’s instructions. Protein levels were normalized with DNA amount of each trephined explant tissue.

### Statistical Analysis

All experiments were repeated at least 3 times, each in duplicate. For IL-1β, TNF-α and MMP-9 detection, each group had three samples at each time point, and each sample was assayed in duplicate. Group means were compared using the appropriate version of ANOVA. Statistical significance was determined using a two-tailed test, where *p* values <0.05 were taken as being significant. Summary data are reported as the mean ± SD.

## Results

### Airlift Culture Induced Squamous Metaplasia of Conjunctival Epithelium

**Figure 1:**
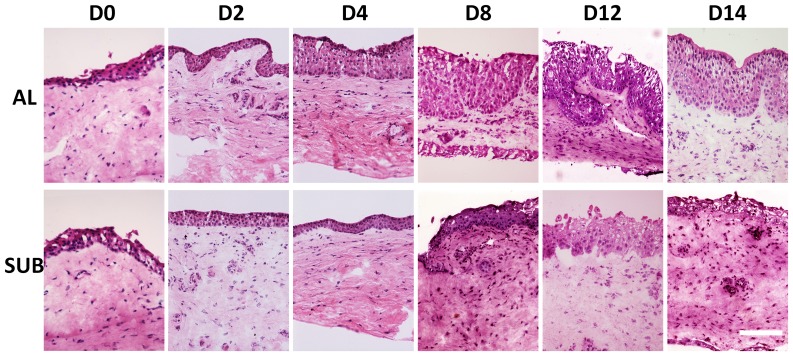
Conjunctival epithelial changes in explant cultures. Hematoxylin & Eosin staining showed increases in conjunctival epithelial cell layers in airlift culture. Their number particularly increased from day 8 to day 14, resulting in an undulated basal epithelial plane and digital invasion. In contrast, the cell layers of submerged culture did not show significant increase. Bar represents 200 µm.

Intact human conjunctival explants containing 3–5 layers were cultured under submerged or airlift conditions for different durations. In the airlift mode, conjunctival epithelial cell layers increased dramatically in thickness from day 0 to day 14. Some areas had more than 12 cell layers, resulting in an undulated basal epithelial plane and digital invasion (Fig. 1). In contrast, the number of cell layers of submerged cultures did not increase significantly. Moreover, the epithelium in some areas decreased to 2 to 3 layers after 14 days of cultivation.

**Figure 2:**
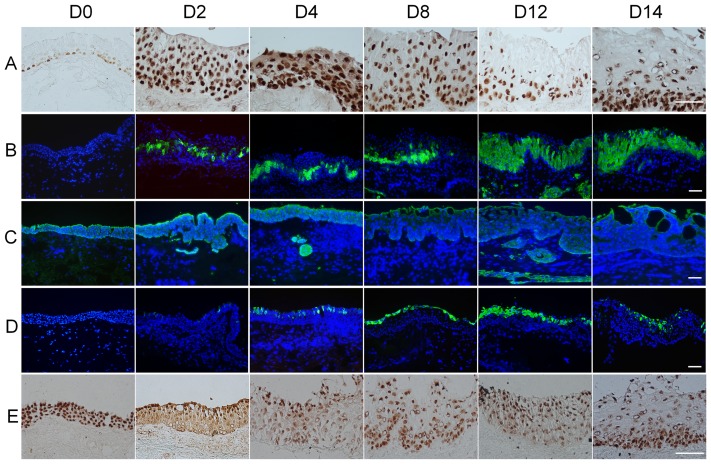
Conjunctival epithelial squamous metaplasia in airlift cultures. (A) P63 staining showed positive nuclei in all basal cells and some suprabasal cells in normal human conjunctiva (D0). In the airlift culture, p63 expression increased throughout the conjunctival epithelial layers at D2, while the superficial cell layers lost p63 expression from D12 to D14. (B) K16 positive cells were not present in normal conjunctival epithelium (D0). In the airlift culture, K16 positive cells emerged in the suprabasal layer from D2 and gradually increased and spread to superficial layers from day 4 to day 14. (C) K19 expression in the full thickness freshly isolated ex vivo conjunctival epithelial cells (D0). K19 expression gradually decreased in the airlift culture after 4 days of cultivation. There were K19 positive cell clusters in the conjunctival stroma. (D) K10 staining was negative in normal conjunctiva before culturing (D0), but became positive in superficial cell layers at day 2 and day 4, and gradually increased to lower layers from day 8 to day 14. (E) Pax6 expressed in full thickness of normal conjunctival epithelial cells (D0), and it decreased in the basal and supra-basal layers in airlift culture from D2 to D14. Bars represent 100 µm.

To determine whether the aforementioned increased stratification of the conjunctival epithelium was due to an increase in cellular proliferation, immunostaining for p63 and K16 keratin was performed. P63 positive cells were noted in most of the basal and some suprabasal layers of normal conjunctiva (Fig. 2A, D0). However, from days 2 to 10, p63-positive cells became evident over the entire epithelium, but thereafter vanished in the superficial cell layers and decreased to a level that was still greater than normal conjunctiva on day 12 and day14 in airlift culture (Fig. 2A). However, conjunctival epithelial cells in the submerged culture did not exhibit any positive p63 nuclear staining in the superficial layers (Fig. S1A). K16 keratin, indicative of an alternative pathway of keratinocyte proliferation, was up-regulated in stratified squamous epithelium showing hyperproliferation or abnormal differentiation [21], was not expressed in normal conjunctival epithelial cells (Fig. 2B, D0). In the airlift cultures, K16 was present in the suprabasal layers at day 2 and gradually increased into the superficial layer cells from day 4 to day 14 (Fig. 2B). In the submerged cultures, although there was positive K16 staining, it was retained in the suprabasal layers (Fig. S1B). Collectively, these results indicate that increased stratification is associated with active proliferation of conjunctival epithelial cells in airlift cultures.

**Figure 3:**
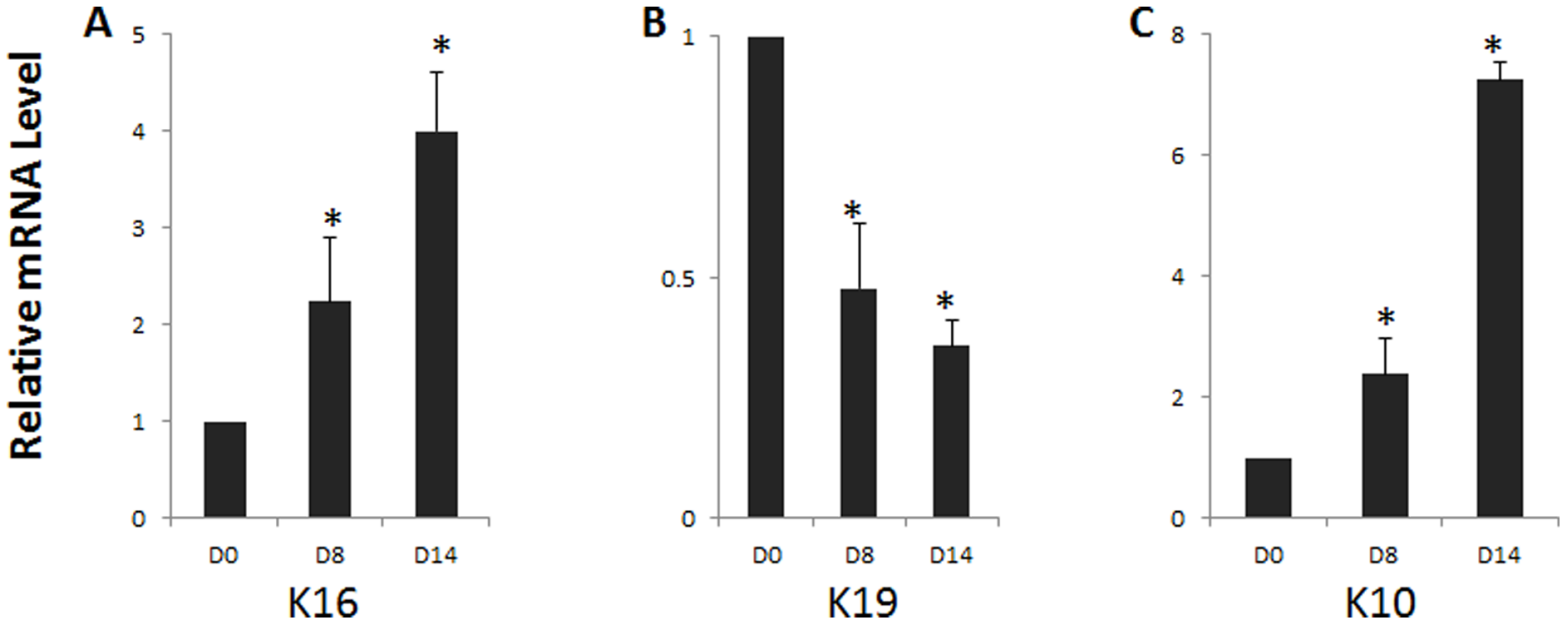
Cytokeratin gene expression level changes in airlift cultures. Realtime PCR showed that K16 (A) and K10 (C) gene expression progressively increased from day 8 to day 14, whereas K19 (B) gene expression significantly decreased on day 8 and day 14 in airlift cultures. *P<0.05.

To investigate whether the conjunctival epithelial phenotype was maintained in airlift cultures, immunostaining was performed for K19 and K10 keratin. As reported, K19 keratin, one of the major conjunctival epithelial cytokeratins [22], was uniformly expressed in all normal conjunctival epithelial cells before culturing (Fig. 2C, D0). However, K19 gradually declined from day 8 in the full-thickness epithelium in airlift cultures (Fig. 2C). In contrast, K19 was well maintained after 2 weeks culture under the submerged condition (Fig. S1C). As expected, K10 keratin, an epidermal keratinocyte-specific intermediate filament, was negative in normal conjunctival epithelium (Fig. 2D, D0). However, in airlift cultures K10-positive cells emerged at day 2 in the superficial cell layer, and gradually increased thereafter from day 4 to day 14 (Fig. 2D). In contrast, there were no K10 positive epithelial cells in all the submerged cultures (Fig. S1D). Quantitative real-time PCR also showed that K16 and K10 were up-regulated, whereas K19 gene expression level was down-regulated on day 8 and even more dramatically on day 14 in airlifting group compared with day 0 controls (Fig. 3A–C). These results indicate that airlift culture also induced abnormal epidermal differentiation in conjunctival tissue.

To determine whether the aforementioned abnormal epidermal differentiation was associated with lineage transdifferentiation in airlift cultures, we performed immunohistochemical staining for Pax6. It is a transcription factor which is distinguishing characteristic of the whole ocular surface epithelium whereas not found in the epidermal epithelium [23]. Pax6-positive nuclei were present throughout the full thickness conjunctival epithelium before culturing (Fig. 2E, D0). In the airlift culture, nuclear expression of Pax6 decreased in the basal and suprabasal epithelial cells from day 2. On the other hand, it became evident in the cytoplasmic and perinuclear regions. This localization pattern gradually spread to superficial layer cells during 2 weeks of cultivation. Interestingly, Pax6 nuclear expression returned to the basal layer at the end of 2 weeks of airlift culture (Fig. 2E, D14). In contrast, in submerged cultures Pax6 nuclear expression was maintained in the full thickness epithelial cells throughout the culture duration (Fig. S1E). Our previous study showed that abnormal epithelial differentiation was highly correlated with down-regulation of Pax6 expression in severe ocular surface diseases [24]. Putting together, air exposure induces down-regulation and loss of Pax6 nuclear staining. Its decline correlates with abnormal epithelial differentiation in ex vivo conjunctival explants.

### Wnt Signaling Pathway Activation in Airlift Cultures

**Figure 4:**
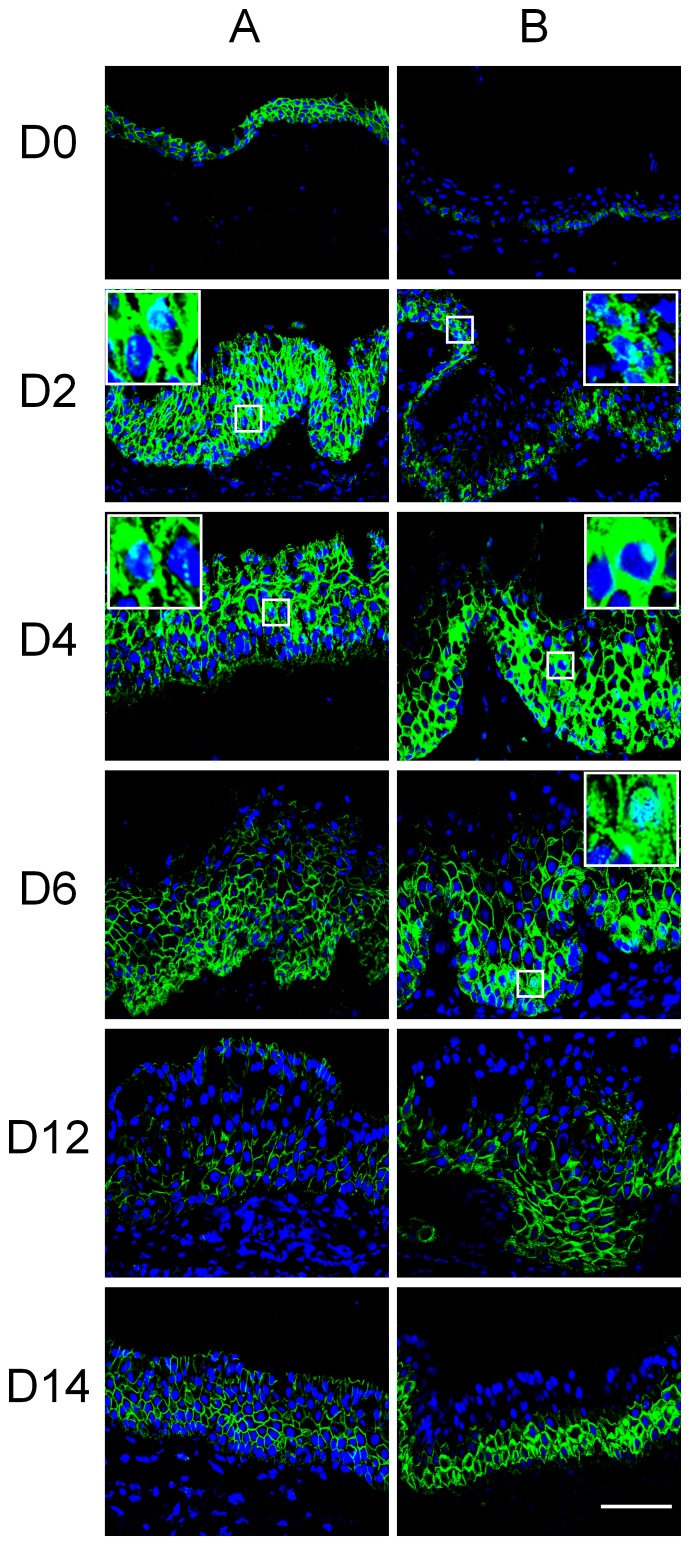
Wnt/β-catenin signaling pathway activation in conjunctival epithelium of airlift explant cultures. β-catenin (column A) and phosphor-β-catenin (Ser-552, column B) staining were performed at different time points after airlifting culture. β-catenin mostly anchored to the cell membrane and showed very low expression levels in cytoplasm of normal conjunctival epithelium (D0). Phosphor-β-catenin was only found in the cell membrane of basal conjunctival epithelium. Cytoplasmic accumulation of β-catenin and phosphor β-catenin in conjunctival epithelium in airlift explants started at day 2, and then gradually decreased after day 6. Nuclear translocation of β-catenin and phosphor β-catenin was seen in the basal and suprabasal layers of conjunctival epithlial cells from day 2 to day 6 (see inserts). At days 12 and 14, β-catenin and the majority of phosphor-β-catenin has translocated to the cell membrane. Inserts are high power magnification of specific locations. Bar represents 100 µm.

Wnt/β-catenin signaling pathway plays an essential role in regulating many cellular processes including cell proliferation, cell differentiation, inflammation, migration, and cell survival [17], [25]. Deregulation of the Wnt pathway is involved in ocular surface neoplasia as well as in other tissues [26]. Phosphorylation of β-catenin at Ser552 by AKT dissociates β-catenin from cell-cell contacts and it then accumulates in both the cytosol and nucleus, which results in increased Wnt/β-catenin pathway activity [27]. To determine whether the Wnt signaling pathway was activated in airlift culture, β-catenin and phosphor β-catenin (Ser552) expression levels were determined at different explant culture stages. The results showed that β-catenin was expressed in the cell membrane and at low levels in the cytoplasm of full thickness epithelium (Fig. 4A, D0), while phosphor β-catenin was only expressed in the basal epithelium of normal conjunctiva (Fig. 4B, D0). In airlift culture, up-regulation, cytoplasm accumulation and nuclear translocation of β-catenin (Fig. 4A) and phosphor β-catenin (Fig. 4B) was prominent from day 2 to day 6, and then gradually decreased and accumulated thereafter at the cell membrane. In submerged culture, accumulation of cytoplasmic β-catenin and phosphor β-catenin was noted on day 8 at a much lower level compared to that of the airlift group (data not shown). Putting together these results, Wnt/β-catenin signaling pathway activation occurs at an early stage of airlift cultures.

### Goblet Cell Lost and Membrane-associated Mucins Alteration in Airlift Cultured Conjunctival Epithelium

**Figure 5:**
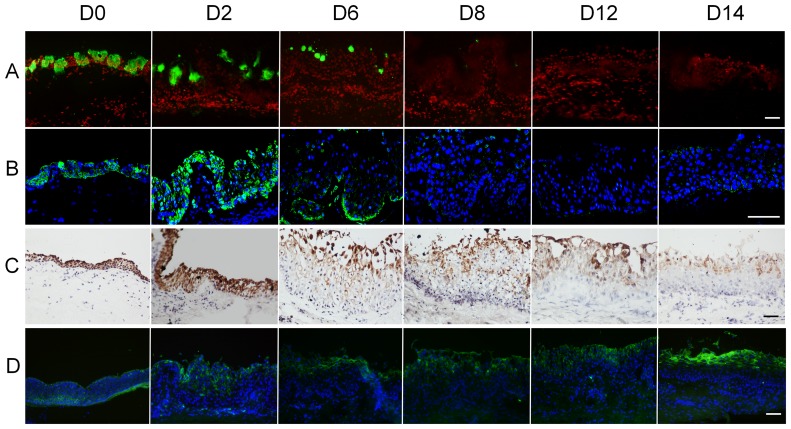
Mucin expression in airlift conjunctival explant cultures. MUC5AC (A) showed scattered expression in normal human conjunctiva, which decreased after 2 days airlift culture, and became negative from day 8 to 14. MUC19 (B) expressed in the full thickness freshly isolated healthy ex vivo conjunctival epithelium. It dramatically decreased after 4 days airlift culture and was undetectable from day 8. MUC4 (C) expressed in the full thickness of epithelial cells in normal conjunctiva. It declined from day 4 in airlift culture. MUC16 (D) expressed in the full thickness of freshly isolated healthy ex vivo conjunctival epithelium. In the airlift group, MUC16 expressed in superficial and some suprabasal epithelial cells from D2 to D14. Its expression was stronger in the superficial squamous shaped cells at day 14. Bars represent 100 µm.

**Figure 6:**
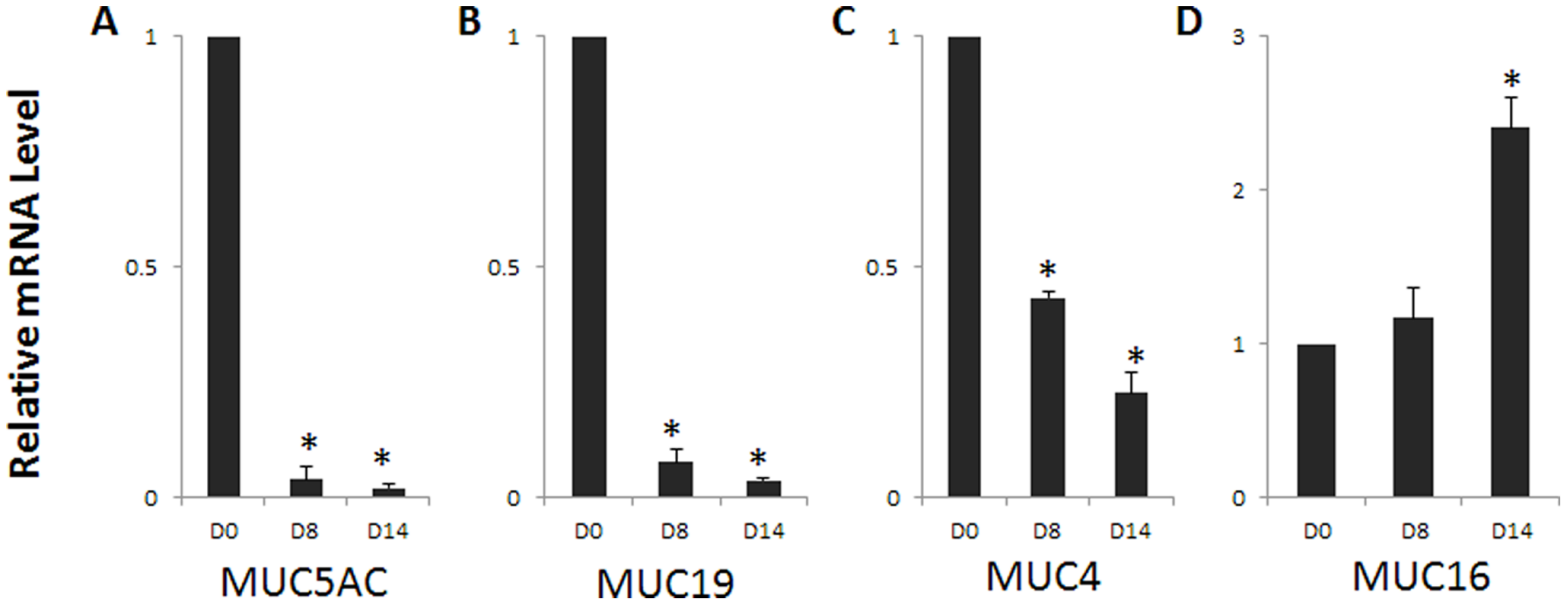
Mucin gene expression level changes in airlift cultures. Realtime PCR showed that MUC5AC (A), MUC19 (B) and MUC4 (C) gene expression significantly decreased on day 8 and day 14, whereas MUC16 (D) mRNA level significantly increased on day 14 in airlift cultures. *P<0.05.

Mucins and their binding partners form an ocular surface barrier and function as signaling molecules (for review, see [28]). Mucin expression declines in dry eye patients [29], [30]. To determine if airlift culture alters mucin expression patterns, MUC5AC, MUC19, MUC4 and MUC16 immunostaining was performed. There was scattered expression of MUC5AC in normal human conjunctiva (Fig. 5A, D0). In airlift cultures, MUC5AC expression decreased from day 2 and was no longer detectable after day 8 (Fig. 5A). In submerged cultures, MUC5AC maintained prominent staining on day 6, gradually declined thereafter and became negative on day 14 (Fig. S2A). MUC19 in airlift cultures had a similar pattern of changes as MUC5AC (Fig. 5B), while only showed mild decrease on day 14 in submerged cultures (Fig. S2B). MUC4 and MUC16, the membrane-bound mucins, lost their full-thickness expression pattern (Fig. 5C and 5D, respectively) and were expressed in the superficial layers in airlift cultures. However, both MUC4 and MUC16 strongly expressed in the full-thickness epithelium at the end of submerged cultures (Fig. S2C and S2D, respectively). Quantitative real-time PCR showed that MUC5AC, MUC19, and MUC4 transcription level were significantly down-regulated on day 8 and day 14, whereas MUC16 gene expression level was up-regulated significantly on day 14 in airlifting group compared with day 0 controls (Fig. 6A–D). These mucins expression patterns in airlift cultures show similar alteration to clinical dry eye [31].

### Air Exposure Induced Apoptosis in Conjunctival Epithelial and Stromal Cells

**Figure 7:**
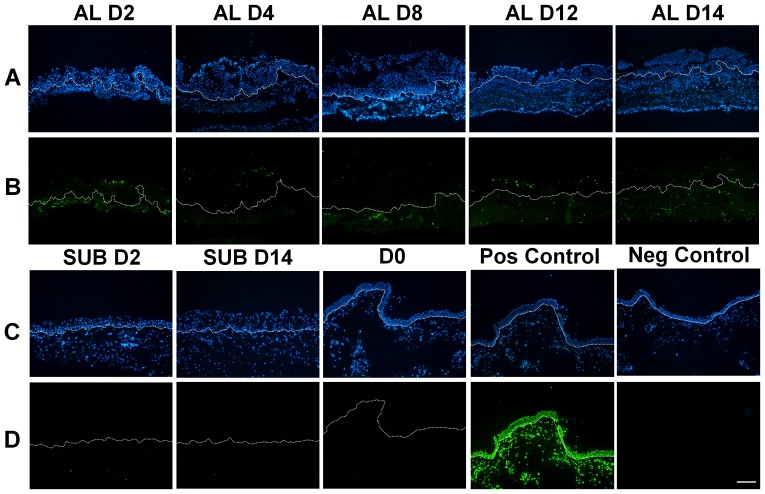
Apoptosis of conjunctival epithelial Cells and stromal cells in airlift cultures. Lanes A and C were counterstained with the nuclear of DAPI dye. Lanes B and D show apoptotic cells based on green fluorescence using the TUNEL assay in the same visual field. Apoptotic cells appeared in both the epithelium and stroma from day 2 to day 14 in airlift cultures, while there were no apoptotic cells in freshly isolated healthy ex vivo conjunctival tissue before culture (D0), and only sporadic apoptotic cells in submerged explants throughout the culture duration (SUB D2 and SUB D14). Bar represents 200 µm.

Normal conjunctival tissue exhibited no apoptosis assessed by TUNEL assay (Fig. 7, D0). In the airlift explants, TUNEL positive cells became evident in both the epithelium and stroma from day 2 to day 14. In contrast, there were only sporadic apoptotic cells in submerged explants throughout the culture duration (Fig. 6).

### IL-1β, TNF-α and MMP-9 Increases in Conditioned Media in Airlift Culture

**Figure 8:**
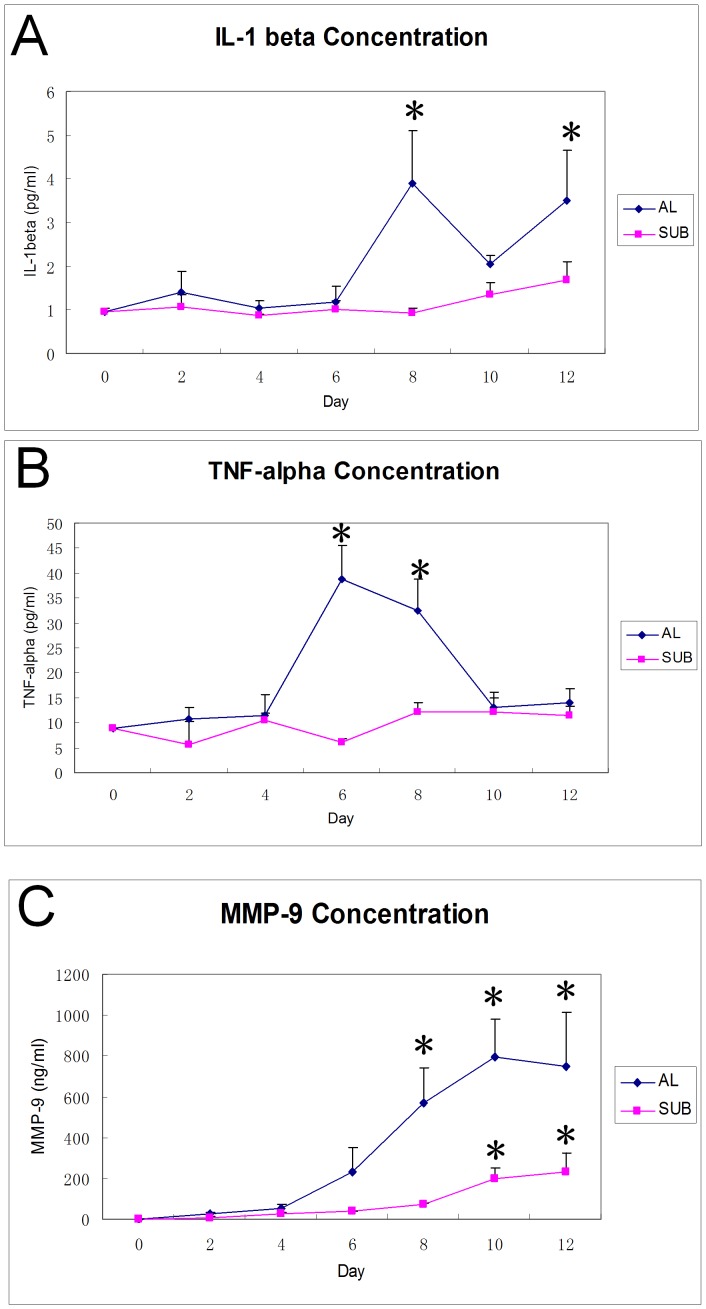
Increased proinflammatory mediators in conditioned media of airlift cultures. IL-1β concentration was maintained at a low level from day 0 (0.97±0.08 pg/mL) to day 6 (1.03±0.16 pg/mL) in airlift culture, while it increased at day 8 (3.89±1.41 pg/mL, P<0.001), and remained high level at day 12 (3.50±1.33 pg/mL, P<0.001). The TNF-α concentration increased after 6 days of airlift culture (8.81±0.26 pg/mL at day 0, 38.71±7.88 pg/mL at day 6, P<0.001) and declined at day 10. MMP-9 remained at a low level from day 0 to day 4, and gradually increased from day 6 to day 12 in airlift culture. (0.27±0.08 ng/mL, 230.61±138.47 ng/mL, 571.78±196.06 ng/mL, 796.49±216.05 ng/mL, and 749.28±307.97 ng/mL in airlift culture for 0, 6, 8, 10 and 12 days respectively). In submerged cultures, no dramatic increases of IL-1β and TNF-α were detected, while MMP-9 only increased significantly after 10 days of culture (195.95±66.07 ng/mL at D10 and 230.99±107.46 ng/mL at D12, P<0.001). However, MMP-9 concentrations remained lower than those in airlift culture from day 6 to day 12 (P<0.001).

In order to investigate whether airlift culture can induce increases in IL-1β, TNF-α, and MMP-9, ELISA measurements on culture media were performed, and the levels were normalized with DNA amounts. IL-1β increased after 8 days of airlift culture and then slightly declined thereafter, however, it remained a relatively high level on day 12 (p<0.001, D0 vs. D8, and D0 vs. D12, Fig. 8A). TNF-α concentration increased after 6 and 8 days in airlift cultures, (Fig. 8B, p<0.001, D0 vs. D6, and D0 vs. D8), but then declined on D10 and D12 (p = 0.344 and p = 0.253 respectively, compared with D0). In contrast, IL-1β and TNF-α levels did not increase at each of these time points in submerged cultures (Fig. 8A–B). MMP-9 dramatically increased on day 8 (p = 0.001, D0 vs. D8) and was sustained at a high level until day 12 (p<0.001, D0 vs. D10, D12, Fig. 8C) in airlift culture. In contrast, MMP-9 remained at a low level for 8 days and then gradually increased from day 10 to day 12 in submerged cultures (Fig. 8C), however, its expression level was still much lower than that in an airlift culture (p<0.001).

## Discussion

We established an ex vivo human conjunctival tissue airlift culture model that simulates many of the described characteristics of dry eye disease. Its features include squamous metaplasia, dry eye related mucin expression profiles, apoptosis and increases proinflammatory cytokine expression levels. These changes occur within 2 weeks in a setting removed from the immune system. This airlift model is simple and easy to handle, and is advantageous for dissecting certain aspects of dry eye pathogenesis.

Air exposure is a common maneuver to induce epithelial stratification in organotypic cultures and tissue-engineering [32], [33]. We found that conjunctival culture exposure to air for 2 days causes squamous metaplasia, evidenced by epithelial cell hyperproliferation and abnormal differentiation. These changes became more prominent around 1 week, similar to our previous study on limbal tissue [19]. As grading of squamous metaplasia correlates well with dry eye severity and the insufficiency of tear film components clinically [34], the airlift model might make it feasible to determine if there is an association between air-exposure level and extent of squamous metaplasia.

Downregulation of Pax6 is associated with abnormal epidermal differentiation in severe ocular surface diseases such as chemical burn, Stevens-Johnson syndrome [24], and other diseases as pinguecula [35] and pterygium (unpublished observation). Downregulation of Pax6 was also found in the rabbit corneal epithelium induced by the embryonic dermis to undergo epidermal transdifferentiation [36]. The present study showed that in conjunctival epithelial airlift cultures, Pax6 down-regulation and loss of nuclear expression started from day 2. These changes support the notion that such declines are related to the abnormal differentiation of conjunctival epithelial cells in our model. Further study using this airlift model may provide us with new insight on the mechanism of abnormal differentiation regarding the function of Pax6.

The Wnt signaling pathway is an important modulator of cell proliferation and differentiation in various cell types. Activation of Wnt pathway is associated with early signs of squamous metaplasia [37], and related to transdifferentiation of non-keratinized mammary epithelium into epidermis [38]. Canonical Wnt pathway activation was also found in FGF-7 over-expression transgenic mice with corneal squamous neoplasia [26]. In a Dkk2 null mouse, lack of a Wnt pathway inhibitor prevented corneal fate decision in the ocular surface epithelium during development, resulting in epidermal differentiation [39]. In our airlift culture, activation of the Wnt pathway happened as early as 2 days and lasted for about 1 week. The associated squamous metaplasia suggests that the Wnt pathway was involved in eliciting epithelial hyper-proliferation and epidermal differentiation. Nevertheless, at a late stage of cultivation, β-catenin relocated to the cell membrane, albeit phosphor-β-catenin remained at a relatively high level compared to healthy non airlifted conjunctiva. Further study is needed to investigate in this model the mechanism of the dynamic Wnt signal activation in squamous metaplasia.

Gel-forming mucins and membrane-associated mucins in the ocular surface are thought to be essential to maintain the integrity of tear film. Gel-forming mucin MUC5AC and MUC19 decline in Sjögren’s syndrome and other types of dry eye [28], [29], MUC5AC was also suppressed in several dry eye animal models [40], [41]. In our study, goblet cell density fell in both airlift and submerged groups, based on less MUC5AC immunostaining and less MUC5AC gene expression level. MUC5AC levels diminished earlier in the airlift than in the submerged cultures. MUC19 also underwent early down-regulation during airlift culture. Membrane-associated mucins such as MUC4 and MUC16 lost their full-thickness expression patterns in superficial layers in airlift cultures, while there was no dramatic change in the submerged culture.

Inflammatory mediators such as IL-1α, IL-1β, IFN-γ and peptidoglycan (PGN) can upregulate MUC16 expression in conjunctival epithelial cell line [42]. Its upregulation occurs in inflammation-associated diseases such as atopic keratoconjunctivitis [43] and Sjögren’s syndrome [44]. In our study, MUC16 expression gradually increased in the apical conjunctival epithelium in the airlift culture, and the mRNA level of MUC16 increased on D8 and D14 after airlifting, which might be due to the increased levels of the aforementioned proinflammatory cytokines. Further study using the airlift model might reveal mucins function in maintaining ocular surface homeostasis.

Increased levels of proinflammatory cytokines and MMPs have been observed in the ocular surface of dry eye patients and various dry eye models [3], [30], [45]–[48]. Our present study provides convincing evidence that conjunctival explants produced proinflammatory mediators such as IL-1β, TNF-α and MMP-9 in response to air exposure for about 1 week, resembling in vivo dry eye pathophysiology. IL-1 and TNF-α can stimulate the production of MMPs by epithelial cells [49]. MMP-9 is also an efficient activator of latent precursor cytokines including IL-1β [50]. Increased MMP-9 activity on the ocular surface in response to dryness can disrupt corneal epithelial barrier function, and partially contribute to membrane-associated mucin expression profile changes [51], [52]. Collectively, our model demonstrated that dryness alone induced production of inflammatory factors without the presence of circulating inflammatory cells. This finding may provide a new way to study the role of inflammatory factors in the pathogenesis of dry eye.

Conjunctival epithelial cell and lacrimal gland epithelial cell apoptosis has been demonstrated in dry eye patients [53], [54] as well as in an animal model [55]. Previous study using an airlifted corneal-limbal recombination culture also described apoptotic cells in the superficial epithelium [56]. In our model, apoptotic conjunctival epithelial and stromal cells were present in airlift explants throughout the culture duration, but not in submerged explants. This indicates that air exposure could induce conjunctival cell apoptosis. Till now the cell signal pathway activation underlying dry eye apoptosis has not yet been elucidated. The proinflammatory ocular surface milieu that develops in dry eye may activate extrinsic apoptosis pathways. Proapoptotic cytokines such as TNF-α and IL-1 have been detected in conjunctival epithelium and tear fluid of dry eye patients [45], and also increased in our airlift culture system. Therefore, we presume that apoptosis might be triggered by air exposure induced dryness alone and/or by rises in inflammatory cytokine levels. Additional studies are warranted to clarify this question.

In conclusion, an air-lifted conjunctival tissue culture is a viable model for characterizing the pathophysiological events associated with dry eye. Its relevance is apparent since many of the hallmarks of this disease can be simulated in this model. Future use of this model may reveal the signaling pathways involved in the pathogenesis of dry eye and epithelial-mesenchymal interactions within this multifactorial disorder. Combining the results obtained with this tissue model and animal models may improve the assessment of new dry eye therapeutic modality.

## Supporting Information

Figure S1 **Conjunctival epithelial squamous metaplasia in submerged cultures.** (A) P63 staining showed positive nuclei in basal and suprabasal cells. Positive cells decreased from day 2 to day 14. (B) K16 was negative at day 2, became positive in suprabasal cells at day 6, and slightly increased at day 14. (C) K19 was expressed in the full thickness epithelium throughout the submerged culture. (D) K10 staining was negative throughout the submerged culture. (E) Pax6 was expressed in the full thickness of conjunctival epithelial cells from D2 to D14. Bars represent 100 µm.
(TIF)

Figure S2 **Mucin expression in submerged conjunctival explant cultures.** MUC5AC (A) showed scattered expression in conjunctival epithelium at day 2 and day 6, and became negative at day 14. MUC19 (B) showed strong expression in the full thickness conjunctival epithelium at day 2 and day 6, while dramatically decreased at day 14. MUC4 (C) showed no significant change throughout the submerged culture. MUC16 (D) expressed in the full thickness of conjunctival epithelium and there was no significant change from day 2 to day 14. Bars represent 100 µm.
(TIF)
