# Supplementary figures and images for: Air Exposure Induced Characteristics of Dry Eye in Conjunctival Tissue Culture
Source: PLoS One. 2014 Jan 31;9(1):e87368. doi: 10.1371/journal.pone.0087368 (PMC3909183; doi:10.1371/journal.pone.0087368)

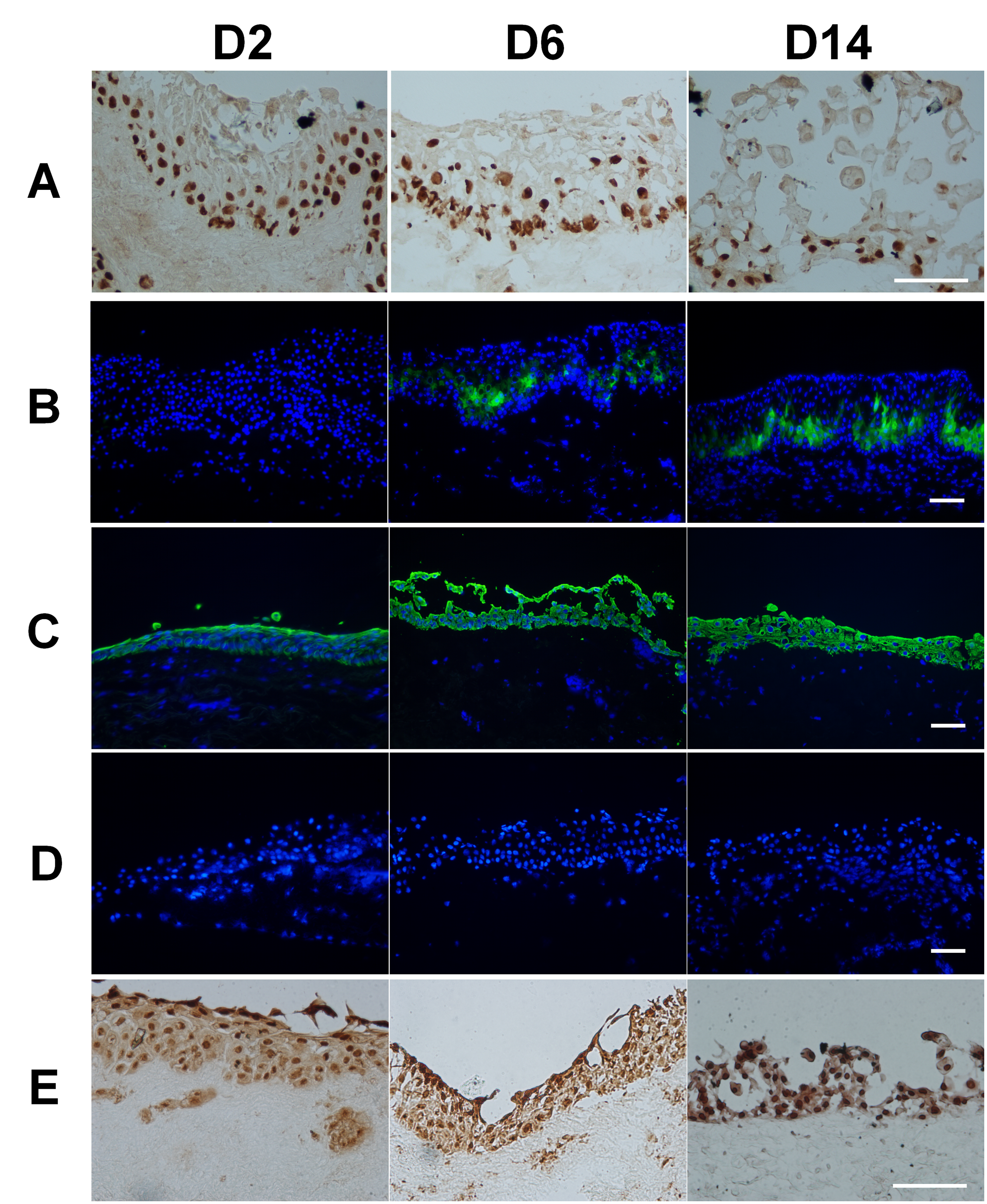

Supplement: Figure S1 — Conjunctival epithelial squamous metaplasia in submerged cultures. (A) P63 staining showed positive nuclei in basal and suprabasal cells. Positive cells decreased from day 2 to day 14. (B) K16 was negative at day 2, became positive in suprabasal cells at day 6, and slightly increased at day 14. (C) K19 was expressed in the full thickness epithelium throughout the submerged culture. (D) K10 staining was negative throughout the submerged culture. (E) Pax6 was expressed in the full thickness of conjunctival epithelial cells from D2 to D14. Bars represent 100 µm. (TIF) [file pone.0087368.s001.tif]

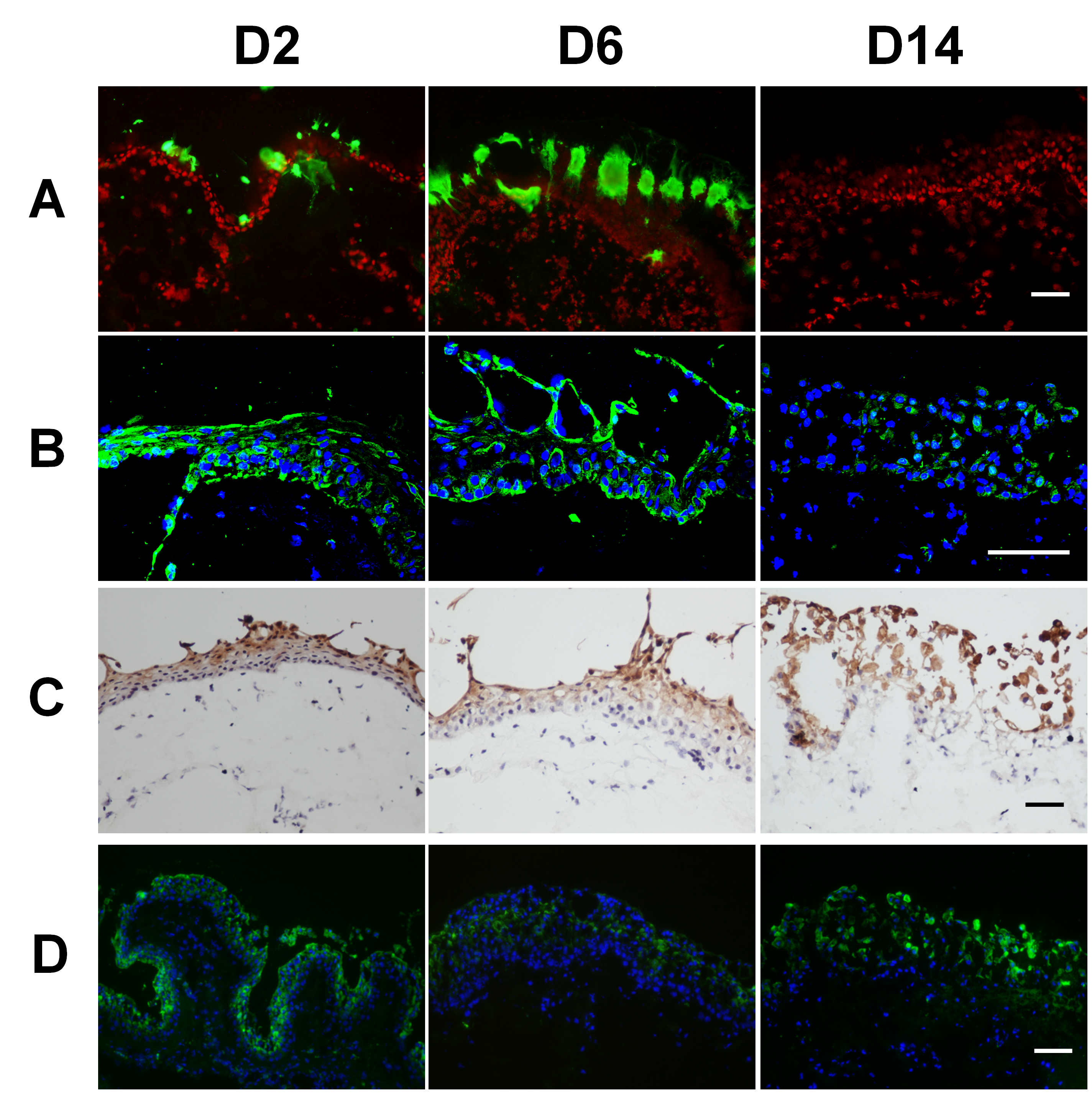

Supplement: Figure S2 — Mucin expression in submerged conjunctival explant cultures. MUC5AC (A) showed scattered expression in conjunctival epithelium at day 2 and day 6, and became negative at day 14. MUC19 (B) showed strong expression in the full thickness conjunctival epithelium at day 2 and day 6, while dramatically decreased at day 14. MUC4 (C) showed no significant change throughout the submerged culture. MUC16 (D) expressed in the full thickness of conjunctival epithelium and there was no significant change from day 2 to day 14. Bars represent 100 µm. (TIF) [file pone.0087368.s002.tif]
